# Effect of Atomic-Temperature Dependence of the Electron–Phonon Coupling in Two-Temperature Model

**DOI:** 10.3390/ma15155193

**Published:** 2022-07-26

**Authors:** Fedor Akhmetov, Nikita Medvedev, Igor Makhotkin, Marcelo Ackermann, Igor Milov

**Affiliations:** 1 Industrial Focus Group XUV Optics, MESA+ Institute for Nanotechnology, University of Twente, Drienerlolaan 5, 7522 NB Enschede, The Netherlands; i.makhotkin@utwente.nl (I.M.); m.d.ackermann@utwente.nl (M.A.); igor.milov@desy.de (I.M.); 2 Institute of Physics, Czech Academy of Sciences, Na Slovance 1999/2, 18221 Prague, Czech Republic; nikita.medvedev@fzu.cz; 3 Institute of Plasma Physics, Czech Academy of Sciences, Za Slovankou 3, 18200 Prague, Czech Republic; 4 Advanced Research Center for Nanolithography (ARCNL), Science Park 106, 1098 XG Amsterdam, The Netherlands; 5 Center for Free-Electron Laser Science CFEL, Deutsches Elektronen-Synchrotron DESY, 22607 Hamburg, Germany

**Keywords:** electron–phonon coupling, two-temperature model

## Abstract

Ultrafast laser irradiation of metals can often be described theoretically with the two-temperature model. The energy exchange between the excited electronic system and the atomic one is governed by the electron–phonon coupling parameter. The electron–phonon coupling depends on both, the electronic and the atomic temperature. We analyze the effect of the dependence of the electron–phonon coupling parameter on the atomic temperature in ruthenium, gold, and palladium. It is shown that the dependence on the atomic temperature induces nonlinear behavior, in which a higher initial electronic temperature leads to faster electron–phonon equilibration. Analysis of the experimental measurements of the transient thermoreflectance of the laser-irradiated ruthenium thin film allows us to draw some, albeit indirect, conclusions about the limits of the applicability of the different coupling parametrizations.

## 1. Introduction

Metals irradiation with ultrashort high-intensity laser pulses is an important tool for both fundamental and applied science. Ultrafast energy deposition into matter drives it into a poorly explored nonequilibrium regime, where unusual material properties and kinetics take place [1,2]. At the same time, it has a broad range of applications such as micromachining, nanotechnology, and materials processing [3,4,5].

Under ultrafast-laser irradiation, a cascade of physical effects takes place, ultimately leading to observable material modifications. Firstly, upon photon absorption, the electronic system of the target acquires a nonequilibrium distribution [2]. During this transient stage, electrons scatter among themselves, thermalizing; their distribution function relaxes to its equilibrium Fermi–Dirac one. Typically, it is assumed that this nonequilibrium stage is short-lived and the electronic ensemble thermalizes at femtosecond timescales. However, in some cases, the out-of-equilibrium state may last for a few hundred femtoseconds up to a picosecond, depending on the excitation level and particular material [6,7].

The energy in the electronic system also dissipates via spatial diffusion outwards from the laser spot in the depth of the material. At the same time, the electrons interact with the lattice via electron–ion (or electron–phonon) scattering. This process transfers the energy absorbed from the laser pulse by electrons to the ionic system of the target.

Phonons, receiving energy from the electrons, can be out-of-equilibrium for even longer times [8]. Relaxation of electrons and phonons and the energy flow between them ultimately define the dynamics of laser-irradiated materials and their final state after irradiation.

The thermal energy flow between electrons and phonons is controlled by the electron–phonon coupling parameter. There were numerous theoretical attempts to calculate this parameter for laser-excited materials [9,10], with the results showing large discrepancies [11]. The electron–phonon, or in a more general way, electron–ion coupling parameter, essentially depends on the current state of the excited material, meaning it is a function of material dynamical variables such as temperature, density, structure, etc. That makes it challenging to calculate and integrate into available models dedicated to simulating laser–matter interaction. 

The most well-known and widely used model that takes into account electron–phonon energy exchange is the two-temperature model (TTM) [12]. In the TTM, a constant or electron-temperature-dependent coupling parameter is typically used [2]. Extensions of the TTM treating different electronic bands and/or different phonon modes separately, each with its own temperature, result in various multi-temperature approaches [13,14]. Such approaches require even more detailed knowledge of the electron–phonon coupling parameter [15].

Apart from theoretical efforts, the tremendous recent development of experimental techniques using ultrafast electron and/or X-ray diffraction enables probing transient states of laser-excited materials with unprecedented spatial and temporal resolution [16,17]. Such methods allow extracting the electron–phonon coupling parameter as a function of irradiation conditions, which can be translated into material dynamical variables using an appropriate theoretical model [8,18]. Such experimental progress stimulates further studies on the electron–phonon coupling parameter which remains one of the least known properties of laser-irradiated materials.

In the previous work, a tight-binding (TB) molecular dynamics (MD) approach to calculate the electron–phonon coupling parameter as a function of electron temperature for various metals across the Periodic Table was used. In the present work, we extend the previous research, focusing on the dependence of the coupling parameter on the atomic temperature. Such dependence was typically ignored in previous works but may be significant at high irradiation doses, as we demonstrate with basic TTM calculations for ruthenium, palladium, and gold elemental metals. We also compare calculations of the coupling parameter using two different parametrizations of the TB part of our model. Finally, we test various calculated coupling parameters on the example of ruthenium using data from our recent optical pump-probe thermoreflectance measurements [19], which allow us to draw some qualitative conclusions [19].

## 2. Model

To study the response of the metallic target to ultrafast irradiation, we employed the two-temperature model [12,20]:

(1) $$\{\begin{array}{c} C_{e}\left(T_{e}\right)\frac{\partial T_{e}}{\partial t}=\frac{\partial }{\partial x}\left(k\left(T_{e},T_{a}\right)\frac{\partial T_{e}}{\partial x}\right)-G\left(T_{e},T_{a}\right)\left(T_{e}-T_{a}\right)+S\left(t,x\right),\\ C_{a}\frac{\partial T_{a}}{\partial t}=G\left(T_{e},T_{a}\right)\left(T_{e}-T_{a}\right). \end{array}$$

Here *T_e_* is the electronic temperature, and *T_a_* is the atomic one, *C_e_*(*T_e_*) is the volumetric electron heat capacity dependent on the electronic temperature (its independence of the atomic temperature will be justified below), *k*(*T_e_,T_a_*) is the electron thermal conductivity dependent on both *T_e_* and *T_a_*, *G*(*T_e_*,*T_a_*) is the electron–ion coupling, *S*(*t,x*) is an external heat source [19] and *C_a_* is the volumetric atomic heat capacity assumed to be constant according to the Dulong–Petit law in the temperature regime we are interested in here.

To study the influence of the electron–ion coupling on temperature evolution in the metals studied, in Section 3 we consider a system to be homogeneously and instantaneously heated by a delta-like laser pulse, serving as the energy source, to the elevated electron temperature *T_e,init_*. In that way, the heat diffusion term can be neglected, and system (1) is solved with the initial conditions:

(2) $$\{\begin{array}{c} T_{e}\left(0\right)=T_{e,init}\;\left(K\right),\\ T_{a}\left(0\right)=300\;K. \end{array}$$

By varying the initial electronic temperature $T_{e,init}$ we studied the electron–phonon relaxation times for equilibrium and kinetics in various metals: ruthenium, palladium, and gold.

Equation (1) requires the knowledge of the parameters *C_a_*, *C_e_*(*T_e_*), *k*(*T_e_,T_a_*) and *G*(*T_e_*,*T_a_*). They need to be provided as external parameters in the model. The atomic heat capacity, *C_a_*, and the electron thermal conductivity *k*(*T_e_,T_a_*) may be found in the literature, e.g., [21,22,23]. To calculate the electron–phonon coupling parameter and the electronic heat capacity, we employed a hybrid code XTANT-3 [24]. The methodology of calculation of the parameters was developed in [11], here we only briefly recall its most essential points. It is based on the combined model of the Boltzmann collision integrals with the TBMD. The transferable TB method allows calculating the transient band structure of the material, the electronic wave functions, and the atomic potential energy surface (interatomic forces required for MD simulations). The overlap of the electronic wave functions with the atomic translation operator provides the probabilities of the nonadiabatic transitions: electron transitions induced by atomic displacements [25,26].

Such calculations provide the transition rates for transitions of electrons between energy levels (band structure) of the materials as a response to atomic displacements. Each atomic displacement results in the evolution of the Hamiltonian of the system, thereby allowing us to construct overlap of electronic wave functions for calculations of the nonadiabatic matrix elements [11]. The calculated matrix elements enter the Boltzmann collision integral. In turn, it enabled us to calculate the energy exchange rate between electrons and atoms, and thus the electron–ion coupling parameter.

The MD simulation traces atomic dynamics in real-time, allowing for any atomic motion—it is not restricted to harmonic oscillations in a perfect periodic structure (phononic approximation of the crystal). Thus, the used method does not imply phononic approximation. Electronic transitions in response to any atomic displacement were calculated, which included an anharmonic atomic motion, such as can be present in melted and/or nonequilibrium systems. Thus, throughout this work, we may use the terms “electron–phonon”, “electron–ion” or “electron–atom” coupling interchangeably.

For the materials studied here, we employed two different transferable TB parameterizations: the Naval Research Laboratory (NRL [27,28]) tight-binding parameterization used in our previous work, and the Density-Functional-based Tight-Binding (DFTB) parameterization [29]. These methods provide the radial functions of the hopping integrals, overlap functions, and repulsive potentials (in the case of DFTB), which allowed us to construct the tight-binding Hamiltonian and calculate interatomic forces for an arbitrary atomic configuration. Both parameterizations employ the Slater–Koster tight-binding scheme with an sp^3^d^5^ linear combination of atomic orbitals (LCAO) basis [27,30]. For ruthenium, we use the DFTB parameters reported in [31], whereas for other metals matsci-0-3 set of parameters is applied [32].

For the calculation of the electronic heat capacity, we used the standard definition via the derivative of the electronic entropy with respect to the electronic temperature [33]. TB calculations provided us with the required electronic band structure.

We performed a series of simulations with various atomic temperatures, *T_a_*, to extract the electron–phonon coupling parameters as functions of *T_a_*. As has been shown in Refs. [11,33], the coupling parameter is nearly linearly proportional to the atomic temperature. Thus, we approximated the dependence of the coupling on *T_a_* with the following relation [33]:

(3) $$G\left(T_{e},T_{a}\right)=G\left(T_{e}\right)\left(1+\alpha \left[\frac{T_{a}}{300K}-1\right]\right),$$

where *G*(*T_e_*) is the coupling parameter dependent on the electronic temperature, and α is the proportionality coefficient to be determined from the TBMD calculations.

## 3. Results

### 3.1. Electron–phonon Coupling Parameter and Electron Heat Capacity

**Figure 1:**
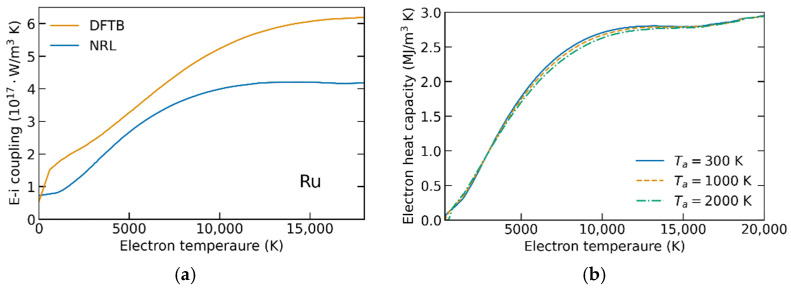
(**a**) Electron–ion couplings *G*(*T_e_*) in ruthenium calculated using NRL and DFTB parametrizations. (**b**) Electron heat capacity in ruthenium calculated for different atomic temperatures.

**Figure 2:**
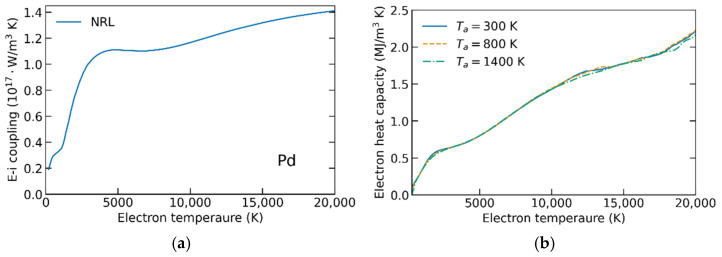
(**a**) Electron–ion couplings *G*(*T_e_*) in palladium calculated using NRL parametrization. (**b**) Electron heat capacity in palladium calculated for different atomic temperatures.

**Figure 3:**
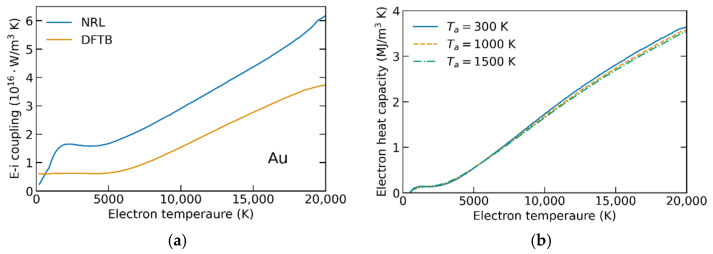
(**a**) Electron–ion couplings *G*(*T_e_*) in gold calculated using NRL and DFTB parametrizations. (**b**) Electron heat capacity in gold calculated for different atomic temperatures.

In a series of XTANT-3 calculations, we extract the electronic heat capacity and the electron–phonon coupling parameter for various electronic and atomic temperatures in ruthenium, palladium, and gold. *G*(*T_e_*)*, C_e_*(*T_e_*) are shown in Figure 1, Figure 2 and Figure 3. The parameters *α* scaling the coupling-parameter dependence on the atomic temperature (entering Equation (3)) are presented in Table 1.

The electron heat capacities were calculated for different atomic temperature values below the respective melting points of the materials. Figure 1, Figure 2 and Figure 3b show that *C_e_* is almost independent of *T_a_*, justifying the omittance of the dependence made above. In all studied materials, the electron heat capacities calculated agree well with other calculations, e.g., [9,23]. This validates our methodology, demonstrating that the used tight-binding models are well capable of calculating the electronic properties of the reported metals.

The electron–phonon coupling parameters’ dependencies on the electronic temperature, calculated with NRL parameterizations, were previously reported in ref. [11], where they were compared to the available experimental data and other calculations. The agreement in gold at high electronic temperatures validated the method [11]. In the current work, the main point is to extend it to the high atomic temperature and analyze its influence on the outcome of the TTM calculations (see the next section).

Additionally, in the two materials for which different TB parameterizations are available (ruthenium and gold, Figure 1 and Figure 3), we analyze the influence of the parameterization on *G*(*T_e_*,*T_a_*). The calculated coupling parameters are noticeably different in both metals—the difference may reach up to 50%. A strong influence of parameterization on the electron–phonon coupling in unexcited materials (at room or cryogenic temperatures) is well-known [11,34]. Here, we confirm that the difference persists in the high-electron-temperature regime. In ruthenium, the DFTB parameterization results in higher values of the coupling than the NRL one, whereas in gold it is the opposite. Thus, we cannot conclude a systematic influence of the TB parameterization, and each material and parametrization requires a dedicated analysis.

### 3.2. The Role of Atomic Temperature Dependence in the Heat Dynamics

**Figure 4:**
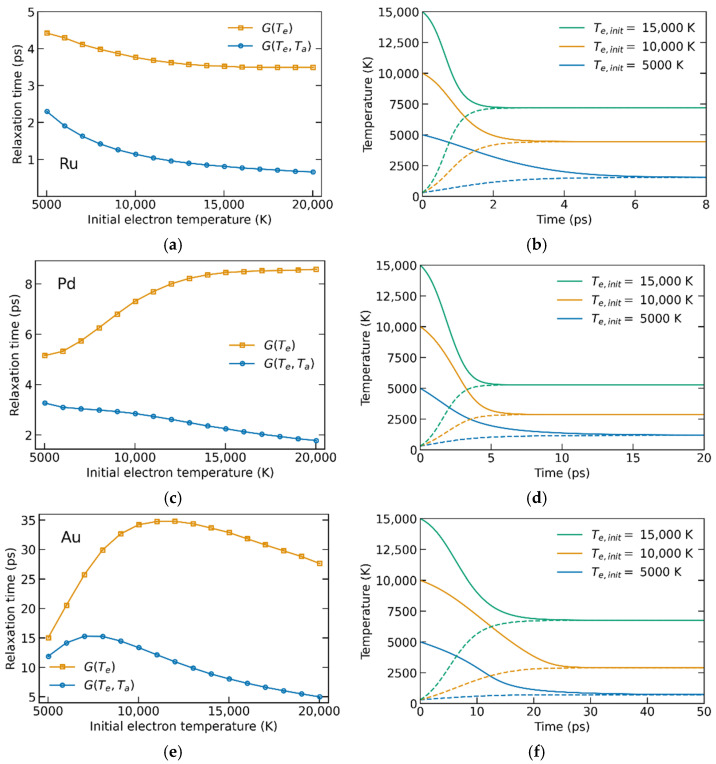
(**a**,**c**,**e**) Electron–ion relaxation times, calculated using both electronic- and atomic-temperature-dependent coupling (blue) and only electronic-temperature-dependent coupling (orange). (**b**,**d**,**f**) Examples of *T_e_*- and *T_a_*-profiles calculated using *G*(*T_e_,T_a_*) for different starting electronic temperatures; solid lines are the electronic temperatures, and dashed lines are the ionic ones. (**a**,**b**) correspond to Ru, (**c**,**d**) to Pd, (**e**,**f**) to Au.

Let us start with the analysis of the influence of atomic temperature on heat dynamics. We consider homogeneously heated metal films with the coupling including the dependence on the atomic temperature *G*(*T_e_*,*T_a_*), and excluding it for comparison, assuming only the electron-temperature-dependent coupling parameter *G*(*T_e_*) *= G*(*T_e_*,*T_a_ =* 300 K). Figure 4 shows the results obtained with the electron–ion coupling calculated using the NRL parametrization. The initial electron temperatures were taken in the range 5 kK–20 kK due to the following reasons: XTANT is unable to provide accurate electron–ion coupling values for the electron temperatures below ~2–3 kK [11]; the upper limit is chosen such that the electron temperature stays far from the plasma limit ($T_{e}\ll E_{F}/k_{B}$) [35], and would not induce significant non-thermal effects such as phonon hardening or considerable electronic pressure [36,37]. Such effects could alter the interatomic potential, and thereby influence parameters of the atomic system, making, e.g., atomic heat capacity and heat conductivity dependent on the electronic temperature. Since these effects play a role only at higher electronic temperatures, it justifies the approximations used for the atomic heat capacity.

Figure 4a,c,e show the electron–ion relaxation times in three considered metals, defined as the moment when the difference between the electronic and the atomic temperatures drops to 1/e from the maximal value. *G*(*T_e_*) results in a much slower equilibration of the electronic and ionic temperatures, as seen in Figure 4.

As follows from Equations (1)–(3), at *t =* 0 (*T_a_*(0) = 300 K) the starting energy exchange between electrons and ions is the same in both cases, with or without the dependence on *T_a_*. After a non-negligible amount of energy is transferred to the ionic system and its temperature increases, the linear term in Equation (3) makes the electron–ion relaxation up to five times faster (e.g., Figure 4a). At high *T_e_* values, *G*(*T_e_*) in Ru and Pd vary slowly and result in the almost constant electron–ion (e–i) relaxation time if the dependence on *T_a_* is excluded, see Figure 4a,c.

The electron–ion relaxation times in gold (Figure 4e) have a pronounced peak at *T_e,init_* around 12 kK (7.5 kK for the case *G = G*(*T_e_,T_a_*)). This peak can be partially attributed to the minimum of *G*(*T_e_*) around *T_e_* = 5 kK (see Figure 3). When *T_e_* decreases from 12 kK to 5 kK, coupling weakens, and energy transfer from electrons to ions slows down resulting in a longer relaxation time. However, at higher initial temperatures two systems exchange a large amount of energy before *T_e_* reaches 5 kK and coupling weakening does not play a significant role. A similar mechanism works in Pd (Figure 4c), but instead of a peak it results in a plateau at *T_e,init_* ≥ 15 kK due to a very slow increase of *G*(*T_e_*) after the minimum.

In contrast, *T_a_*-dependence of the coupling leads to decreasing relaxation with increasing *T_e,init_* for all of the considered metals. Figure 4b,d clearly demonstrate that with an increase in the initial electronic temperature, the equilibrium is reached faster. In Figure 4f this effect is less pronounced due to the above-discussed minimum of *G*(*T_e_*) in gold, but still can be found in a comparison of profiles corresponding to *T_e,init_* = 10 kK (orange lines) and *T_e,init_* = 15 kK (green lines). Such a nonlinear effect is only observed if the dependence of the coupling parameter on the phonon temperature is taken into account. It indicates its importance for modeling materials’ response to ultrafast irradiation, as was also recently noted in ref. [38].

It Is expected that a fast phase transition from solid to a molten state should ensue, which can be directly measured in, e.g., ultrafast diffraction experiments. This result suggests that electron–ion coupling relaxation time should be observable, which could elucidate the role of the atomic temperature dependence of the coupling parameter and validate our calculations in future dedicated experiments.

### 3.3. The Role of Parametrization

Now, let us consider the effect of the chosen TB parametrization on the temperature kinetics on the example of homogeneously heated ruthenium and gold films.

**Figure 5:**
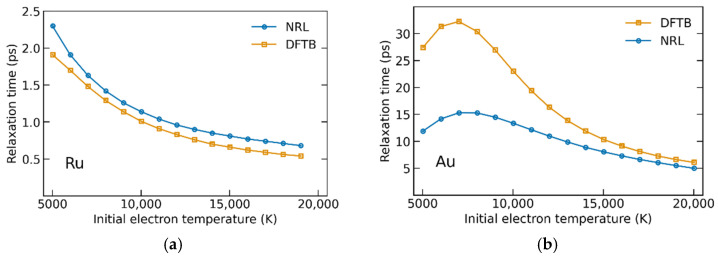
(**a**) Electron–ion relaxation times in ruthenium (**a**) and gold (**b**), calculated using two different TB parametrizations: NRL (blue) and DFTB (orange).

Figure 5a shows electron–ion relaxation times in ruthenium with atomic-temperature-dependent coupling parameter *G = G*(*T_e_,T_a_*) calculated with NRL and DFTB parametrizations. In this case, relaxation times weakly depend on the chosen parametrization. One could expect a divergence between parametrizations at *T_e,init_* > 10 kK as follows from Figure 1a, but the difference in *T_e_*-dependent coupling parameters is suppressed by the dependence on *T_a_*, which is stronger for NRL parametrization. At the initial electron temperatures around 19 kK (this temperature is equivalent to the absorbed energy density of *E_abs_* = 3.46 eV/atom via the relation $E_{abs}=\int C_{e}dT_{e}$) the relaxation time reaches values as small as 0.5 ps.

Electron–ion relaxation times in gold (Figure 5b) demonstrate a strong dependence on the parametrization at low and intermediate values of *T_e,init_*. At high *T_e,init_* > 15 kK, the choice of the parametrization has a smaller impact on the relaxation times. This follows from two factors. First, the overall difference between the coupling parameters decreases with the increasing electronic temperature as soon as hot electrons behave like a free-electron gas (high *T_e_*). Second, the larger value of *α* for DFTB parametrization results in the approaching of DFTB-calculated coupling to NRL-calculated one with the increasing atomic temperature. Both effects lead to similar values of the coupling parameters at high electronic and atomic temperatures.

The results show that the electron–phonon relaxation times in Ru are almost independent of the chosen parametrization. In contrast, the relaxation times in Au are rather sensitive to the tight-binding parameterization used for the calculation of the coupling parameter in the regime of low and intermediate electron temperatures. A similar conclusion was recently drawn from the analysis of electroconductivity in warm dense aluminum [39].

The electron–ion relaxation time in elemental gold may vary by a factor of two in certain cases, proportionally to the differences in the coupling parameter. This strong difference may be detectable in well-controlled experiments, which should allow for validating the parameterization applicability to calculations of the coupling parameter. We will discuss possible experiments that could provide access to the coupling parameter in the next section.

## 4. Discussion

Direct experimental measurement of the electron–phonon coupling parameter in the highly-excited matter is a very complex task. Unambiguous measurements would require a simultaneous tracing of the electronic and atomic temperatures with femtosecond resolution, which so far has not been achieved. The most advanced techniques at present measure only the transient atomic temperature with the help of the ultrafast electron or X-ray diffraction [13,40,41]. Such methods require state-of-the-art large-scale facilities and are thus extremely rare. Alternative simpler methods of accessing electron–phonon coupling at high electronic temperatures are in high demand.

One of the possible ways to measure electron–phonon coupling in laser-excited materials is based on transient thermoreflectance experiments [42]. In such experiments, the ultrashort pump beam brings a target into a highly nonequilibrium state between the electronic and the phononic systems. The probe beam comes to the target with a variable delay and generates a transient thermoreflectance signal providing information about heat dynamics in a studied target. This signal is then fitted by the temperature profiles taken from the TTM simulations with a variable electron–ion coupling (see e.g., [43,44]). In such a fitting procedure, usually, the reflectance dependence on temperatures is either taken in a model approximation, e.g., Drude model, or assumed to be linearly dependent on *T_e_* and *T_a_* [45]:

(4) $$R\left(T_{e},T_{a}\right)=a\Delta T_{e}+b\Delta T_{a}.$$

In the case of transition metal Ru with a half-occupied d-band that we consider in this section, the Drude model is not able to provide reliable temperature-dependent optical properties because it does not account for the interband optical transitions between d- and s-bands. Possible extensions of the Drude model that account for interband transitions, e.g., the multi oscillator Drude–Lorenz model [46], require a priori unknown parameters, usually extracted from the fitting of a model to optical constants calculated via the computationally demanding DFT-MD approach. Thus, in this work, we use the second methodology, applying Equation (4). We perform an inverse analysis: having various parametrizations for the electron–ion coupling, we calculated *T_e_* and *T_a_* profiles in TTM (1) with electron thermal conductivity taken from [23] and fitted the thermoreflectance signal from our recent pump-probe experiment on Ru thin films [19] by varying the coefficients *a* and *b* in Equation (4).

We compared three parameterizations for the electron–ion coupling in Ru: *T_e_*- and (*T_e_*, *T_a_*)- dependent couplings as presented in this work, and *T_e_*-dependent coupling calculated by Petrov et al. [23]. Petrov et al.’s work uses a different methodology than ours, which relies on the Eliashberg formalism of the electron–phonon coupling parameter calculation. Their required parameters—the band structure and equilibrium phonon spectrum (spectral function)—are extracted from the density functional theory calculations. As was discussed in ref. [11], Eliashberg formalism was developed for low-temperature, superconducting conditions, and its extension to high electronic temperatures proposed in ref. [47] and now used in many works including ref. [23] is questionable.

We took a thermoreflectance signal measured on a 30 nm Ru/Si sample irradiated by 85 fs 800 nm laser pulse with 31 mJ/cm^2^ incident fluence [19]. Under our experimental conditions, the electron temperature change exceeds 4000 K and the atomic temperature change is ~1000 K. Although linear dependence between the reflectance and temperatures, Equation (4), strictly speaking, is only valid at small temperature changes, on the order of a few hundreds of Kelvins, we could still achieve a satisfactory fit to our data in our qualitative analysis using such linear dependence.

**Figure 6:**
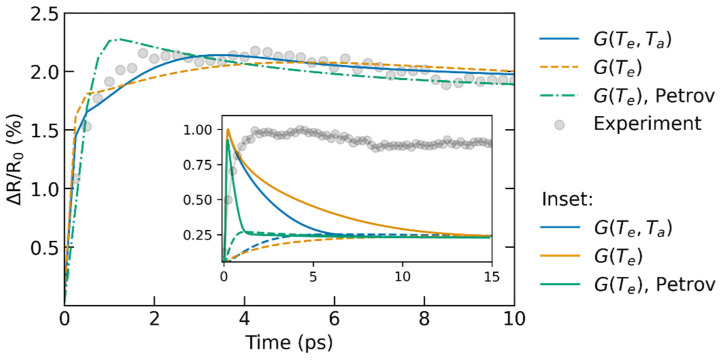
Thermoreflectance data fitted by Equation (4). Temperature profiles were calculated using three electron–ion coupling parametrizations: coupling with linear *T_a_*-dependence (1) (blue solid line), the same coupling without linear to *T_a_* term (orange dashed line), and *T_e_*-dependent coupling provided by Petrov et al. [23] (green dash-dotted line). The inset shows normalized electronic and atomic temperature profiles calculated with TTM vs thermoreflectance data. Solid lines correspond to *T_e_*, dashed ones to *T_a_*.

The experimental data demonstrated in Figure 6 allow for distinguishing three different processes: (i) The initial rapid increase within 0.5 ps is expected to be associated with the excitation of the electronic system; (ii) The slow increase from ~0.5 ps up to the maximum at ~3 ps is the result of the atomic heating via the electron–ion coupling; (iii) The decrease after ~3 ps is associated with cooling due to heat transport out of the laser-irradiated spot.

Let us point out that the rapid change in the reflectance at t < 0.5 ps may be strongly affected by nonequilibrium within the electronic system. In a nonequilibrium state, the electronic system does not adhere to the Fermi–Dirac distribution, and the electronic temperature is ill-defined. This limits the applicability of the TTM and the analysis with the help of Equation (4). We thus focus our analysis on the time window from ~0.5 ps to 3 ps, where the thermoreflectance change is mainly affected by the atomic temperature and thus the electron–ion coupling.

The results of fitting show that the available couplings do not describe the entire heat dynamics at the same level of accuracy. Petrov et al.’s coupling can reproduce heat dynamics at timescales ≤ 1 ps due to the very fast equilibration of electronic and atomic temperatures, making fitting insensitive to *T_e_*. In contrast, the coupling *G*(*T_e_,T_a_*) reported in the present work provides a better agreement at longer timescales, from ~0.5 ps onwards, during the essential electron–phonon coupling and later cooling. We thus conclude that the calculated electron–phonon coupling *G*(*T_e_,T_a_*) provides the best fitting to the experimental data in the region where TTM is expected to be applicable, which may serve as its qualitative validation.

Unfortunately, the pump-probe thermoreflectance measurements do not allow us to unambiguously conclude which coupling parametrization is more accurate. Although Petrov et al.’s coupling is in good agreement with the experimental coupling measured at room temperature [23], it does not fit well with the data in Figure 6. In contrast, XTANT simulations seem to underestimate coupling at low electronic and atomic temperatures, but, as follows from the provided results, provide a better agreement with the experiment under intermediate excitation. We think this discrepancy is a result of the limited sensitivity of thermoreflectance to the dynamics of the electronic and atomic systems: at the very first ps after the excitation, the probe cannot discriminate a contribution of each system into the signal. We also note here that Petrov et al.’s coupling was previously used to calculate the ablation of Ru in good agreement with the experiment [48].

The definitive answer to the question of which coupling is better may be addressed in experiments probing the dynamics of excited electrons and atoms separately. Such an experiment could be, e.g., a combination of ultrafast electron/X-ray diffraction (probing the atomic system) and optical thermoreflectance spectroscopy or more sensitive EUV absorption spectroscopy (probing the electronic system independently) [49].

## 5. Conclusions

We present calculated electron–phonon coupling parameters in Ru, Pd, and Au as a function of both electronic and atomic temperatures. For all of the considered materials, we demonstrated that the atomic-temperature-dependent coupling has a great impact on the electron–ion relaxation time for equilibrium at intermediate and high absorbed doses. It is, thus, important to take into account the dependence of the coupling parameter on the atomic temperature for reliable simulations.

We also provide a comparison of the electron–phonon coupling in Au and Ru calculated with two different transferrable tight-binding parametrizations. We find that in Au the choice of parametrization plays an important role in electron temperatures below ~15 kK, which has implications for the analysis of ultrafast laser–matter interaction experiments.

Finally, we present the analysis of transient thermoreflectance from Ru thin films using different electron–phonon coupling parametrizations and demonstrate that our calculations, with atomic-temperature dependence included, provide a good agreement with the experimental data available. It further emphasizes the importance of accounting for the atomic temperature in calculations of the coupling parameter.

## Figures and Tables

**Table 1:** Parameter *α* of the linear dependence of the coupling on *T_a_* in Equation (3).

Material	α	
	NRL	DFTB
Ru	0.55	0.45
Pd	0.55	-
Au	0.45	0.65

## Data Availability

The data produced in this study are available from the authors upon reasonable request.
